# A Survey on Urban Traffic Management System Using Wireless Sensor Networks

**DOI:** 10.3390/s16020157

**Published:** 2016-01-27

**Authors:** Kapileswar Nellore, Gerhard P. Hancke

**Affiliations:** Advanced Sensor Networks Research Group, Department of Electrical, Electronic and Computer Engineering, University of Pretoria, Pretoria 0002, South Africa; gerhard.hancke@up.ac.za

**Keywords:** Wireless Sensor Networks (WSNs), traffic sensing systems, intelligent traffic light controllers, emergency vehicle priority, traffic congestion, Average Waiting Time (AWT), traffic parameters

## Abstract

Nowadays, the number of vehicles has increased exponentially, but the bedrock capacities of roads and transportation systems have not developed in an equivalent way to efficiently cope with the number of vehicles traveling on them. Due to this, road jamming and traffic correlated pollution have increased with the associated adverse societal and financial effect on different markets worldwide. A static control system may block emergency vehicles due to traffic jams. Wireless Sensor networks (WSNs) have gained increasing attention in traffic detection and avoiding road congestion. WSNs are very trendy due to their faster transfer of information, easy installation, less maintenance, compactness and for being less expensive compared to other network options. There has been significant research on Traffic Management Systems using WSNs to avoid congestion, ensure priority for emergency vehicles and cut the Average Waiting Time (AWT) of vehicles at intersections. In recent decades, researchers have started to monitor real-time traffic using WSNs, RFIDs, ZigBee, VANETs, Bluetooth devices, cameras and infrared signals. This paper presents a survey of current urban traffic management schemes for priority-based signalling, and reducing congestion and the AWT of vehicles. The main objective of this survey is to provide a taxonomy of different traffic management schemes used for avoiding congestion. Existing urban traffic management schemes for the avoidance of congestion and providing priority to emergency vehicles are considered and set the foundation for further research.

## 1. Introduction

Over the years vehicle usage has increased exponentially worldwide. Due to this, road traffic conditions have become complicated and chaotic. According to the Victoria Transport Policy Institute’s Urban Mobility Report (UMR) dated 18 December 2014 [1], huge amounts of time and money are wasted, and e.g., time delay: 5.5 billion hours and fuel wasted: 2.9 billion gallons in urban areas of the United States due to traffic congestion between 2000 and 2010. The UMR predicted that congestion cost will increase from $121 billion (in 2011) to $199 billion (in 2020). Accidents occur at road intersections due to malfunctions in Traffic Light Control Systems (TLCSs) and drivers’ negligence. By improving the operations in traffic management systems, safety and efficiency of transportation systems will increase. A common traffic control system utilizes static signalling times at intersections and does not provide priority to emergency vehicles such as ambulances, firefighters and police cars, possibly causing a loss of lives, damage or destruction of property, and increased fuel costs, pollution and congestion. An Intelligent Traffic Management System aims at managing traffic effectively during emergencies through the use of cutting-edge communication and processing technologies and appropriate intelligent system algorithms.

In the present state-of-the-art, a wireless sensor network is a promising technology that offers a solution for the design and development of a good deal of traffic control system applications. The sensor network consists of a sensor and gateway nodes. The duty of the sensor node is to monitor traffic in an allocated area, utilizing different devices that can measure several physical traffic parameters like flow, density, volume, headway, waiting time, throughput, as well as pollution. The gateway node collects the traffic information from all the nodes and directs the same to the base station.

**Figure 1:**
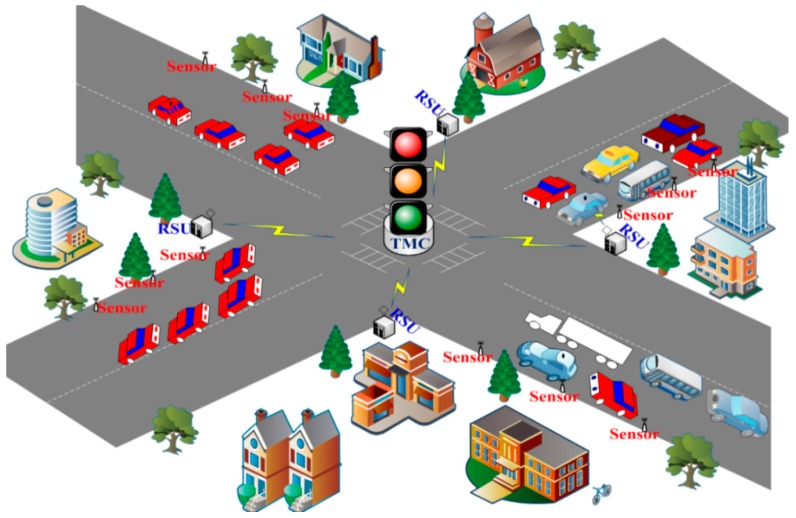
WSN-based Urban Traffic Management System.

The WSNs have attracted wide interest from academic and industrial researchers due to their lower maintenance, low price, and usage in a wide-ranging application areas, such as health, the military, industry and the home. The exclusive characteristics of WSNs include the mobility of sensor nodes, the ability to withstand harsh environmental conditions, node failures, low power and scalability.

**Figure 2:**
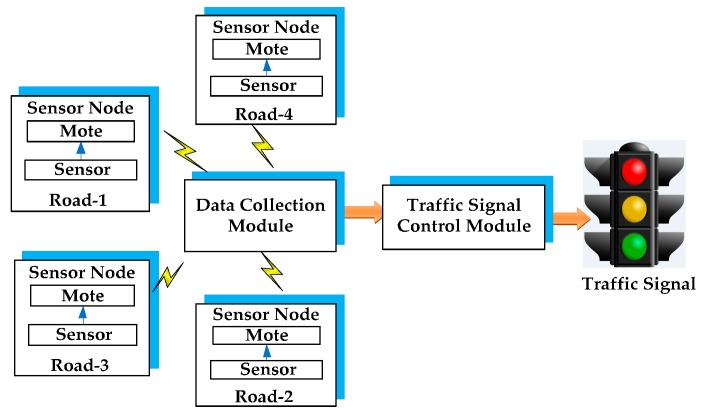
Schematic of a traffic management centre.

A typical WSN-based Urban Traffic Management System (W-UTMS) is depicted in Figure 1. A scattered W-UTMS application achieves four functions: (i) information collection; (ii) data diffusion; (iii) processing of data to plan the required activities; and (iv) implementation of the suitable actions. To carry out these functions autonomously, the UTMS is equipped with wireless sensors, a Traffic Management Centre (TMC), a Road Side Unit (RSU) and On Board Units (OBUs) on vehicles. Sensors collect the real-time traffic information, like vehicle density, type of vehicle, average waiting time and pollution and relay the traffic data to the RSU. When an emergency vehicle approaches the intersection, the OBU directs that information to the RSU. The RSU gathers the data from all sensor nodes and OBUs and forward the same to the TMC. The schematic of a typical TMC is presented in Figure 2. 

The data collection module of the TMC gathers the data, analyses the traffic parameters and then sends it to the traffic signal control module of the TMC. The control module evaluates the data and takes intelligent decisions to offer signalling priority for emergency vehicles. After the passage of an emergency vehicle, the system resumes its regular operation. This system will optimally manage the urban traffic congestion, the priority for emergency vehicles, waiting time of vehicles, fuel consumption, and safety. 

Lately, Vehicular Sensor Networks (VSNs) have been proved as a great solution for monitoring traffic. In VSNs, vehicles are equipped with sensing devices and these vehicles move about the city to sense the traffic. Vehicles transfer the sensing data to a city traffic monitoring centre by using vehicle-to-vehicle or vehicle-to-infra wireless communications. The vehicular sensing system has a high coverage capability and low deployment cost. Dynamic traffic monitoring using vehicular networks (DTMon) is an alternative technique to collect reliable information about traffic in free-flow and in transient-flow conditions. 

The Average Waiting Time (AWT) for a vehicle at an intersection in a conventional system is unpredictable. Schemes that are smart and adaptive to traffic flow are continuously being developed and used for building an Intelligent Traffic Management System (ITMS), which can decrease the AWT of vehicles at the intersection and regulates the traffic flow. 

This paper provides a complete review of existing techniques for WSN-based urban traffic management, discusses key challenges and identifies future research directions. The remaining part of this paper is organized as follows: in Section 2, an overview of the key issues is given. Section 3 provides sensing evolution and traffic sensing technologies. Section 4 reviews some related work on urban traffic management schemes for priority based signalling, reducing congestion and AWT of vehicles. Section 5 highlights various challenges of Urban Traffic Management Systems. Section 6 concludes the paper by providing future directions.

## 2. Key Issues in Urban Traffic Management System

Traffic congestion is a burning issue in many cities due to an exponential growth of running vehicles. There are basically two types of traffic congestion. The first one is recurring traffic congestion, which appears at the same place during the same time every day. The second one is non-recurring traffic congestion, which occurs randomly like an unplanned event. This non-recurring effect can cause a sudden traffic volume increase. Detection of non-recurring traffic congestion is critical compared to the recurring type, because it requires real-time traffic information and evaluation thereof with appropriate traffic management decisions.

**Figure 3:**
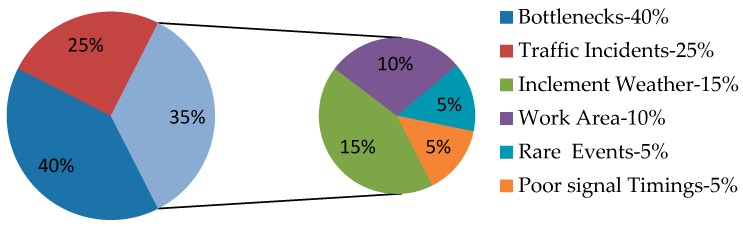
Sources of urban traffic congestion.

**Figure 4:**
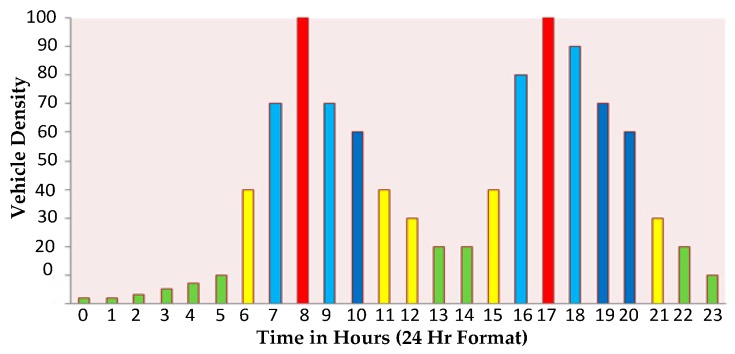
Urban traffic density.

Congestion is mainly due the inadequate capacity of the roadways to efficiently move the number of traveling vehicles on them. To activate the congestion relief process, first we have to determine the sources of congestion. A US Federal Highway Administration report [2] defines six sources of congestion. Figure 3 shows the congestion sources and their contribution. The points where the roadway narrows are termed “bottlenecks”. Vehicle crashes and stalls are termed “traffic incidents”. Road repairs, building of new roads and maintenance activities are termed as “work area”. “Inclement weather” such as excessive rains, snowfall and fog cause congestion. “Poor signal timing” occurs when the traffic light controller is faulty and there is no relation maintained between time allocation for a signal and traffic volume. “Rare events”, e.g., strikes and marathons, cause a surge in traffic volume and resulting congestion. An estimated graph of everyday traffic density is shown in Figure 4. On the X-axis, time in 24 h format is shown and on the Y-axis, vehicle density is considered. In the early hours, *i.e.*, from 1h00 to 5h00, vehicle density is at its lowest as most people sleep at night. The vehicle density increases exponentially and reaches a maximum value at 8h00 as it is considered peak hour when people go to the office, school and college. The vehicle density decreases gradually between 10h00 and 14h00. The vehicle density again increases exponentially and reaches a maximum value at around 17h00 as people return back home. The vehicle density decreases gradually between 20h00 and 24h00. Therefore, the peak hours are usually between 8h00 to 9h00 and 17h00 to 18h00. Most of the congestion occurs at this time and is recursive. Because of traffic incidents and rare events, the traffic density may increase additionally. Designing an adaptive and dynamic traffic control system to provide smooth traffic flow in peak busy hours can be an interesting issue for future research.

To prevent congestion in urban and metropolitan areas, the design requirements to be considered in developing a new traffic management system are:

- Hierarchical road infrastructures for public transportation.
- Reliable information on real-time traffic should be provided to users and traffic management systems.
- The traffic control system should be fast in taking decisions.
- The highest priority to the emergency vehicles at intersections to save lives and property.
- The system has to detect road accidents.
- A smart city traffic system should provide security.

## 3. Overview

This section provides an overview of the sensing evolution, traffic sensing technologies, the characteristic of the general sensor node and the hierarchical functionality of a WSN-based urban traffic management system. 

### 3.1. Sensing Evolution

The Sensor is a key element of any smart system and a course of action is taken based on its location. The control system gathers the data from a group of sensors and uses different variables to distinguish its location and modifies its actions consequently. The accessibility of a massive amount of various sensors and endlessly growing technology facilitates applications that were unfeasible in the earliest because of high prices and restricted handiness. Technological developments have driven the improvement behind sensors and also powered the small-scale devices by making use of the sensors at a low price. From the viewpoint of the desires of smart traffic management, an extensive handiness of the technology transforms to a great amount of chances in the sensing.

### 3.2. Traffic Sensing Technologies

The safe and efficient operation of a traffic management system relies largely on the application of advanced technologies. As a result, the past decade has witnessed the wide application of communication, sensing and computing technologies in traffic management, event detection, emergency response, fleet management and travel assistance. There is a requirement for effective traffic organization, to avoid congestion and optimize traffic flow at intersections. An approach to control traffic flow is to make use of sensor technologies. Table 1 lists a few types of traffic sensing technologies that are frequently employed in traffic surveillance for data collection. The table also lists the advantages and disadvantages of present sensor technologies in terms of installation, maintenance, performance in various atmospheric conditions and varying traffic flow [3]. Table 2 lists the output data, coverage and bandwidth requirements of each sensor technology. 

### 3.3. General Sensor Node

A sensor is a transducer which transforms the physical nature parameters like light, temperature, velocity, pressure, moisture, *etc*. to an electronic signal. This electronic signal can be understood by humans or fed into a control system. A traffic monitoring sensor node typically comprises of four main modules as given below:

- A sensing module—This module acquires data.
- A processing and storage module—This module process the local data and stores it.
- A radio module—This module is for wireless data communication.
- A power module—This module is for energy supply.

A general sensor node normally includes a radio module for wireless data communication. The transmission range of wireless communication depends on the communication technology, which can be a few meters (Bluetooth, Zig Bee, Wi-Fi, *etc.*) to thousands of kilometers (Wi-MAX, GSM, *etc*.). The wireless communication has numerous technologies and standards, including Zig Bee, Bluetooth, GPRS, GSM, Wi-Fi and Wi-MAX [4]. Table 3 highlights the frequency, range, throughput and feature of these wireless communication technologies.

### 3.4. Hierarchy of Urban Traffic Management Systems

**Figure 5:**
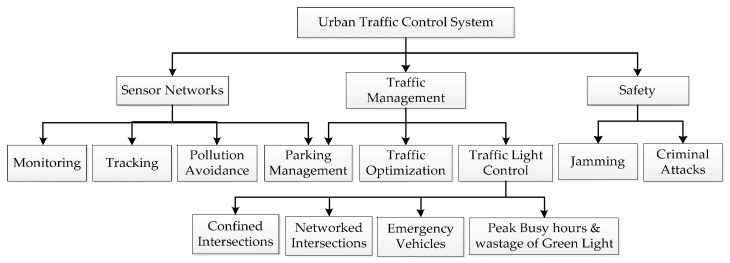
Hierarchical functionality of WSN based urban traffic management system.

The urban traffic management system is mainly divided into three subsystems, namely the sensor network subsystem, the traffic control subsystem and the safety subsystem. The sensor network subsystem collects real-time traffic information and quickly directs that to the traffic control subsystem, which manages the traffic congestion for normal and emergency vehicles at intersections by using adaptive traffic algorithms. The last subsystem provides security to the wireless traffic control system against jamming and violation attacks. We can classify the functions of TMS into three categories, *i.e.*, congestion avoidance, prioritizing emergency vehicles and reducing Average Waiting Time (AWT). The hierarchical functionality of WSN based urban traffic management system is shown in Figure 5.

**Table 1:** Overview of traffic sensing technologies [3].

Technology	Principles	Advantages	Disadvantages	Specific Equipment
Inductive loop	The inductive-loop sensor detects the vehicle or conductive metal object by sensing the loop inductance, which is dropped by inducing currents in the object.	Flexible design to fulfill a great variety of applications. Unresponsive to bad weather. Offers accurate count data.	Installation and maintenance require pavement cut and lane closure. Many loops are required to cover a location. The detection accuracy drops with vehicle classes.	Roadway sensors, lead-in cables, pull box and electronic unit in the control cabinet.
RFID (Radio-frequency identification)	RFID technology uses radio waves to give-and-take data between a reader and an electronic tag attached to a vehicle for the purpose of tracking.	RFID is economical. It does not disturb traffic.	RFID only senses equipped vehicles at a point on the road.	Antenna (transmitter and receiver), Transponder, tag reader system, and computer.
Microwave radar	The Microwave radar transmits signals in the recognition regions and captures the echoed signals from vehicles. The reflected signal is processed to find the speed and direction of the vehicle.	Unresponsive to bad weather. Speed is measured directly. Multiple lane operation.	Continuous wave Doppler sensors are incapable of sensing immobile vehicles.	Antenna (transmitter and receiver), control unit and processor.
Acoustic	Acoustic sensors detect audible sounds produced by vehicular traffic and there by vehicle presence, and speed are measured.	Unresponsive to precipitation. Multiple lane operation.	Vehicle count accuracy may be affected by cold temperature.	Transducer, filters, microphones, pre amplifier, storage equipment.
Magnetometer	Magnetometers have sensors that sense the horizontal and vertical components of the Earth's magnetic field.	Less prone than loops to pressures of traffic. Unresponsive to bad weather. Data transmission over RF (Radio Frequency) link.	Installation needs a pavement cut. Inadequate installation decreases pavement life cycle. Maintenance and installation require lane closure.	Magnetic probe detector, micro loop probes and control unit.
Magnetic	A magnetic sensor detects the presence of a vehicle by measuring the perturbation in the Earth's magnetic field because of a ferrous metal object.	Applicable where loops are not likely. Installation of some models does not require a pavement cut. Insensitive to bad weather. Less prone than inductive loops to pressures of vehicles.	Installation needs a boring under the road. Incapable of sensing immobile vehicles.	Magnetic probe detector, micro loop probes and control unit.
Infrared	The infrared sensor illuminates the low powered infrared energy in the recognition regions and captures the echoed energy from the vehicles. The echoed energy is focused onto an infrared-sensitive material, which transforms the echoed and illuminated energy into electrical signals. These signals are processed and analyzed to obtain the presence of a vehicle.	The vehicle information such as speed, position and class are accurately measured by the transmission of multiple beams. Multiple lane operation.	Sensitive to bad weather. Installation, maintenance and lens cleaning require lane closure.	Multi spectrum camera.
Aerial/Satellite Imaging	This technology involves the use of either manned or unmanned helicopters in the sky to capture imageries of the ground and the imageries are transmitted to a workspace for investigation.	Traffic surveillance can be taken at high accuracy. It is a non-intrusive and non- interruptive technology. It can offer a bird’s eyesight of the system-wide traffic situations.	Helicopters are expensive and necessitate pilots to operate. It costs time and resource to gather traffic data. Analysis of aerial pictures is complicated.	Helicopters, Analog color PAL camera and computer.
Ultrasonic	An Ultrasonic sensor transmits ultrasonic waves and again collects the echoed waves from an object. It uses the time lapse between the transmitted and reflected sonic wave to identify the location of the object.	Monitors multiple lanes. Proficient of detecting over height vehicles.	Performance is affected by environmental circumstances. Occupancy measurement on freeways may be degraded with large pulse repetition periods.	Transducers (Transmitter and Receiver), amplifier and oscillator.
VIP (Video image processor)	This system normally consists of a camera, processor-based workstation for analyzing the images, and software for understanding the imageries and transforming them into traffic data.	Monitors multiple lanes. Simple to add and change detection areas. Offers broad-area detection.	Installation and maintenance require lane closure. Performance is sensitive to bad weather, vehicle shadows, and dusts on the camera lens. Requires specific camera mounting height for finest vehicle presence detection and speed measurement.	Analog color PAL camera and image processing unit.

**Table 2:** Traffic output data and communications bandwidth of commercially available sensors [3].

Technology	Vehicle Count	Presence	Speed	Output Data	Classification	Multiple Lane, Multiple Detection Zone Data	Communication Bandwidth
Inductive loop	**✔**	**✔**	✔ *	**✔**	**✔ &**		Low to modest
Magnetometer	**✔**	**✔**	✔ *	**✔**			Low
Magnetic induction coil	**✔**	**✔ $**	✔ *	**✔**			Low
Microwave radar	**✔**	**✔ #**	**✔**	**✔ #**	**✔ #**	**✔ #**	Moderate
Active infrared	**✔**	**✔**	**✔ @**	**✔**	**✔**	**✔**	Low to modest
Passive infrared	**✔**	**✔**	**✔ @**	**✔**			Low to modest
Ultrasonic	**✔**	**✔**		**✔**			Low
Acoustic array	**✔**	**✔**	**✔**	**✔**		**✔ ^**	Low to modest
Video image processor	**✔**	**✔**	**✔**	**✔**	**✔**	**✔**	Low to high

* Two sensors can be used to measure speed; & With specific electronics device that categorizes vehicles; $ By using distinct sensor layouts and data processing software; # By using a microwave radar sensor and suitable signal processing unit; @ With multi detection region; ^ By suitable beam forming models and data processing unit.

**Table 3:** Wireless Communication Technologies [4].

Technology	Description	Standard	Frequency	Range	Throughput	Feature
Wi-MAX	Standard for data transmission via radio waves.	IEEE 802.16	2–11 GHz	<10 km	<75 Mbps	High speed and serve number of users.
ZigBee	Specification of a set of complex wireless communication protocols for use with low consumption digital radios, based on WPAN standard IEEE 802.15.4.	IEEE 802.15.4	2.4 GHz	<75 m	250 Kbps	Mesh networks, Multiple protocol availability.
Bluetooth	Standard for data and voice transmission between many devices via a safe and free radio link.	IEEE 802.15.1	2.4 GHz	Class 1: 100 m Class 2: 15–20 m Class 3: 1 m	v. 1.2:1 Mbps v. 2.0:3 Mbps UWB: 53–480 Mbps	Low power version available.
UWB	UWB is merely a radio technology that can be used as part of an overall standard.	IEEE 802.15.3a	3.1–10.6 GHz	10 m2 m	110 Mbps480 Mbps	Extremely fast transfer of files between servers and portable devices.
Wi-Fi	System of wireless data broadcast over computational webs.	IEEE 802.11a; 802.11b/g/n	5.8 GHz 2.4 GHz	<100 m	11/54/300 Mbps	High speed and ubiquity.
GSM	Typical system for communication via mobile phones including digital technology.	--	850/900/1800/1900 MHz	Dependent on service provider	9.6 Kbps	Large coverage, High capacity and transmission quality.
GPRS	Extended GSM for packet data communication.	--	850/900/1800/1900 MHz	Dependent on service provider	56–144 Kbps	High resource utilization, Short access time.
RFID	Uses radio waves to detect objects carrying tags.	--	125 KHz, 13.56 MHz, 902 to 928 MHz	Up to 3 m	9.6–115 Kbps	Low cost.

## 4. State-of-the-Art Review

This section provides a complete review of related projects, architectures, data collection schemes, routing algorithms, congestion avoidance schemes, priority based traffic management schemes, and average waiting time reduction schemes on urban traffic management, based on WSNs.

### 4.1. Related Projects

Considerable research has been done on Traffic Management Systems using WSNs for congestion avoidance, prioritizing emergency vehicles and reducing Average Waiting Time (AWT) of vehicles at intersections. During the last years, quite a lot of projects on urban traffic management have been developed focusing on different traffic parameters. They are presented in Table 4. 

A $423 m Intelligent Transport System (ITS) [5] was developed to improve the traffic management and control system on Hong Kong’s road network, which is one of the busiest road networks in the world. This project was started in 2001 and effectively completed in 2010. The key platforms included in this project to address traffic congestion are traffic organization, monitoring, data analysis and control activities. This project has ensured optimal traffic control by tracking of all the main highways, trunk roads and road tunnels.

Sridhar *et al.* [6] designed and tested a low-cost sensor network instrument for monitoring traffic in a work zone. They started this project in 2009 with funding of £30,144.00 and completed it in 2010. A complete sensor network system collects data in work zones and presents it for post facto analysis and to the internet. 

Al-Holou *et al.* [7] formulated the vehicle impact on the environment, jamming and traffic safety as a multi-dimensional model. They started this project with a budget of $80,064 in 2010 and completed it in 2012. The adaptive sign control application recommended in this project is directed to attain two main goals: (1) improving traffic flow and diminishing traffic density; (2) refining traffic safety at intersections. An adaptive traffic light controlling approach, which uses V2V/V2I communications epitomize a revolution in the traffic management area.

The main attention on modeling and simulation of traffic flow via Fast Lane [8] is to do the computations both fast and with a perfect outcome. Fast Lane is a software tool that can be used for short term estimation of traffic flow on freeways. A superior case study was conducted to show that the framework can be used to predict real-time traffic in the future.

A project titled “Advanced weather responsive traffic management strategies” was started in 2012 with funding of £206,856.00 [9]. The main goal of this project was road weather management. The project was successfully completed in 2013.

The development of an adaptive traffic signal control system (ACS Lite) for Wolf Road, Albany, NY, USA [10] started in 2012 with funding of £569,800.00. The objectives of this project are to demonstrate the Siemens ACS-Lite technology and signaling efficient system at the junctions along Wolf Road in Albany, NY. This project team includes URTC member Rensselaer Polytechnic Institute (RPI) and the City College of New York (CCNY) and non-UTRC members Siemens ITS, SenSys Networks, and Annese and Associates, Inc. The project was successfully completed in 2013.

**Table 4:** Urban Traffic Management Projects.

Project Name	Objectives	Project Sponsor	Year of completion
Hong Kong ITS project [5].	To perform an optimal traffic management.	Hong Kong Government.	2010
A Distributed Instrument for Measuring Traffic in Short-Term Work Zones [6].	To design, construct, and test a low-cost sensor network instrument to monitor traffic in work zones.	Research and Innovative Technology Administration, US	2010
A Multi-Dimensional Model for Vehicle Impact on Traffic Safety, Congestion, and Environment [7].	To use technology for creating a safe, efficient and greener environment.	Research and Innovative Technology Administration, US	2011
Fast Lane: modelling and simulation of traffic flow [8].	Prediction of the traffic flow.	Dutch traffic and transport laboratory for students, Dutch	2013
Advanced Weather Responsive Traffic Management Strategies [9].	To perform road weather management.	Research and Innovative Technology Administration, US	2013
Adaptive Traffic Signal Control System (ACS Lite) for Wolf Road, Albany, New York [10].	To dynamically adjust signal timing to meet current traffic demands.	New York State Department of Transportation	2013
Advanced Traveler Information System (ATIS) for Indian Cities [11].	To provide congestion information, alternate route, travel time and alert travelers about any accident.	Department of Electronic and Information Technology (DeitY), India	2014
Agent- Based Traffic Management and Reinforcement Learning in Congested Intersections [12].	To minimize travel time and reduce stoppage.	Research and Innovative Technology Administration, US	Start date: 2010-10-01 (In Progress)
A Proof-of-Concept and demonstration of a High Definition, Digital Video Surveillance and Wireless Transmission System for traffic Monitoring and Analysis [13].	To monitor and analyze the traffic through high definition video surveillance and broadcast system.	Research and Innovative Technology Administration, US	Start date: 2009-03-20 (In progress)

To ease the traffic congestion problem, an Advanced Traveler Information System (ATIS) was jointly developed by the Indian Institute of Technology, Madras and C-DAC (Trivandrum), India in 2014 [11]. The network selected for the Real-time Traffic Information System (RTIS) prototype project was a 16 km long stretch that includes heavy traffic roads. Over 100 GPS devices have been positioned on city buses on the route and 32 video cameras are mounted along the roads to collect traffic data. The information to travelers will be provided through Variable Message Sign (VMS) boards on the route and the RTIS website. This project is sponsored by the Department of Electronic and Information Technology (DeitY), Government of India.

Rahim *et al.* [12] started developing an agent-based traffic management technique with reinforcement learning principles in 2010. The Research and Innovative Technology Administration, Washington is the sponsoring organization, which funded the project with £117,786.00. This project ensured that the adaptive operated traffic signals cut down the travel time, fuel consumption and also helps to smooth flow of traffic. This advanced research will lead to developments in the mobility and reliability in the region.

Christopher *et al.* [13] demonstrated a high definition video surveillance and broadcasting system at the University of Maryland campus. This project will allow analysis of video technology for traffic analysis and a deployable wireless image transport system. The Research and Innovative Technology Administration, Washington is the funding organization for this project.

### 4.2. Specific Architectures, Data Collection Schemes and Routing Algorithms

To gratify the requirements for real-time traffic management, researchers have proposed a number of explicit architectures, data collection schemes and routing algorithms for WSN-based urban traffic management. They are reported in the literature.

**Figure 6:**
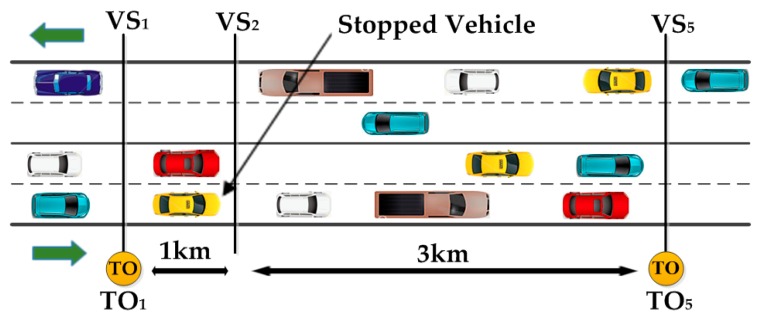
Location of TOs and virtual strips from the entry of a four lane highway [14].

Dynamic Traffic Monitoring using vehicular networks (DTMon) has been used to collect high quality travel time in [14,15]. The main components of DTMon architecture are: Task Organizers (TOs) and Virtual Strips (VSs). DTMon uses the Task Organizers (TOs) to interconnect with moving vehicles. Virtual Strips (VSs) are the traffic data collection points on the roads. The location of TOs and VSs from the entry of a four lane highway is shown in Figure 6. The vehicle rigged with a communication module that communicates with the VS and Traffic Monitoring Control (TMC). They examined the DTMon’s ability using VANET modules. By assigning various tasks and with various locations, the Message Reception Ratio (MRR) and Information Reception Ratio (IRR) are evaluated. The simulation results show better performance of DTMon than an Automatic Vehicle Location (AVL) system in terms of monitoring. The use of virtual strips in DTMon can be extended for detecting and tracking the end-of-queue, caused by congestion.

An investigation of daily changeability in public transport journey time using a Global Positioning System data set for bus travel times can be found in [16]. Short range vehicle-to-vehicle (V2V) and vehicle-to-roadside (V2R) communications in a vehicular environment are found in [17,18]. WAVE/IEEE 802.11p technology is used for V2V and V2R communications. Field trial outcomes demonstrated the improved performance using the advanced radio.

Bruno *et al.* [19] proposed two efficient data collection algorithms: GREEDY and Probabilistic Data Collection (PDC) for vehicular multimedia sensor networks. They simulated the proposed algorithm using a VANETMobiSim and NS-2 simulator. The simulation results show that the GREEDY solution can achieve a more uniform coverage and consume less network bandwidth.

Chao *et al.* [20] proposed an intelligent traffic management system based on RFID for determination of traffic flow. The proposed intelligent traffic light control system (ITLCS) uses an RFID system, which complies with the IEEE 802.11p protocol to detect the number of vehicles and find the time in seconds spent by vehicles on main roads and on side roads passing through the intersection throughout a period of green light. They used Zig Bee modules to send real time data like weather conditions and the vehicle registration information to the regional control centre. The proposed system can perform remote transmission and reduce traffic accidents. 

Saqib *et al.* [21] presented a novel application to estimate the position and velocity of a vehicle using WSNs. They used anchor sensor nodes as roadside readers and when a moving vehicle comes in between the operating range of these two nodes, position information is given by a symmetric double-sided two-way ranging algorithm. By measuring position at different times, the velocity of a moving vehicle is calculated. The proposed model is less expensive compared to other models for detection of position and velocity of moving vehicles. A WSN cross-layer design method has been proposed in [22] comprising the physical, MAC, and network layers for achieving energy efficiency and improving latency.

Choi *et al.* [23] developed an optimal routing policy in the VSN. They studied the important factors that influence the delay performance in VSNs. They formulated the packet routing problem as a Markov Decision Process (MDP) and developed an Optimal VSN Data Forwarding (OVDF) algorithm. They conducted simulations on a GloMoSim simulator. They compared the Performance of OVDF with the Vehicle Assisted Data Delivery (VADD) algorithm and Trajectory Based Data (TBD) forwarding scheme. The simulation results demonstrated that the OVDF gives best sensing coverage, a higher packet delivery ratio and reduces the delay about 25% and 20% compared to VADD and TBD, respectively. Lee *et al.* [24] developed a middleware called MobEyes, which supports vehicular sensor network based urban traffic monitoring applications. They validated MobEyes in a challenging track application.

Friesen *et al.* [25] developed a complete data gathering system at the University of Manitoba, Winnipeg, MB, Canada. The developed system employs a variety of wireless networking technologies and devices to gather inferred traffic data at an intersection along a main street in an urban setting. Vehicle presence and vehicle trajectory information is collected by a Bluetooth device discovery and the information is transmitted to the master node via the IEEE 802.15.4 protocol. The master node handles all incoming data. For real time information about the intersection, the master node sends the data to the server every 5 mins via cellular GSM communication. The server stores the information in the data base, which is accessed by the consumer and corporate websites to get the correct data in real time. They proved Bluetooth tracking as a useful means of capturing vehicular traffic for real-time traffic monitoring.

Liu *et al.* [26] proposed a fast and effective approach to detect and locate a vehicle logo. The logo in the image is located using a Hough transform based algorithm, a texture analysis algorithm and a gradient algorithm. After locating the logo, feature extraction and classification is done by using the scale invariant feature transform, gradient operators and wavelet transform. The experimental results showed the effectiveness of the proposed method.

A user-customizable urban traffic information collection scheme based on WSNs was proposed in [27] to achieve the balance between the energy consumption of sensors and the delay time of data delivery by using all routing attributes for decision-making.

Ahmad *et al.* [28] proposed a channel switching techniques to reduce communication overhead and energy consumption. The proposed model was simulated in OPNET. They observed that their model is feasible for implementation at intersections. However, the proposed model gave negative results of intra-vehicle channel interference. All representative architectures, data collection schemes, and routing algorithms that were analysed in detail are highlighted in Table 5, where data is sorted by author names and reference numbers.

**Table 5:** Summary of architectures, data collection schemes and routing algorithms.

Reference	Proposed Approach	Outcome
Arbabi *et al.* [14]	Dynamic traffic monitoring system.	Collection of high quality travels time and speed.
Mazloumi *et al*. [16]	GPS based tracking system.	Provides shortest route. Traveling time of vehicle reduces.
Bazzi *et al*. [17] Alexander *et al*. [18]	Vertical distance vector routing algorithms for timely data acquisition in VSNs.	Provides highly reliable communication. Maximum coverage range.
Bruno *et al.* [19]	Data collection (Greedy & PDC) Schemes for urban monitoring applications.	Reduced redundancy information. Consumes less network bandwidth.
Chao *et al*. [20]	RFID based intelligent traffic flow control system.	Remote transmission. Traffic accidents are reduced. Effectively control traffic flow.
Saqib *et al*. [21]	Symmetric double sided two way ranging algorithm.	Position and velocity of a moving vehicle are determined with less computation.
Cabezas *et al*. [22]	WSN cross layer design approach to coordinate the transfer of packets.	Latency and jitter are improved.
Choi *et al*. [23]	Delay-optimal VSN routing algorithm (OVDF).	Improved delivery performance of data packets in VSN.
Friesen *et al*. [25]	Prototype of a cost effective Bluetooth traffic monitoring system.	Monitoring vehicle density and traffic directionality. Low power consumption.
Liu *et al*. [26]	Vehicle-logo location algorithm.	Classification of vehicles.
Zhou *et al*. [27]	User customizable data-centric routing.	Fast traffic information delivery.

### 4.3. Congestion Avoidance Schemes

Traffic congestion is a plight situation on roads that occurs as use increases. It is characterized by slower vehicle speeds, queuing, and longer trip times. To reduce congestion, several WSN-based schemes and techniques have been developed and described in the literature. 

Du *et al.* [29] proposed a traffic monitoring and estimation system for Shanghai using VSNs. They proposed two methods: circuit patrol and greedy patrol control algorithms to improve the performance of matrix completion (MC) based traffic monitoring. Simulation results have shown that the proposed algorithms reduced the traffic estimation error from 35% to 10% compared with the random patrol method. Vehicle-to-vehicle communication also helps in reducing traffic congestion [30]. Mobility management methodologies and recent progress in mobile WSNs are discussed in [31].

Dragoi *et al.* [32] presented a congestion avoidance model for traffic control over a vehicular *ad-hoc* network created between the sensors and cars in traffic. The road side wireless equipment (also called wireless traffic lights, WTLs) collects the data from cars in different road segments and accumulates it to form a road map and its costs. They evaluated the proposed model using a VNSim simulator. Their evaluation results showed that the average time desired for the vehicle to reach its endpoint recorded a significant decrease of up to 40%.

Ahmad *et al.* [33] developed a test bed for the evaluation of traffic signal control algorithms using a microscopic traffic simulator (SUMO) and AVR micro-controller. They implemented four scheduling algorithms, *i.e.*, the shortest remaining processing time (SRPT), the Fair SRPT, the minimum destination distance first (MDDF) and minimum average destination distance first (MADDF) in SUMO, studied their effect on traffic networks and measured their execution times. Their experimental results indicated the execution time is constant and independent of traffic intensity for SRPT, decreases with increase in traffic intensity for Fair SRPT, increases rapidly with traffic intensity for MADDF and increases with traffic intensity for MDDF, but not as rapidly as MADDF.

**Table 6:** Summary of congestion avoidance schemes.

Reference	Proposed Approach	Outcome
Du *et al.* [29]	Circuit patrol and Greedy patrol algorithms to improve the estimation of traffic matrix.	The traffic estimation error is minimized from 35% to 10%. Traffic monitoring is improved.
Knorr *et al.* [30]	VANET based strategies for improving traffic state estimation.	Traveling time sinks from 22% to 12% compared with the case without communication. Penetration rates are slightly improved.
Dragoi *et al.* [32]	Traffic model based on the use of cars to collect traffic data and several wireless traffic lights.	Travel time reduced up to 40%. The emission decreased.
Ahmad *et al.* [33]	A test bed for evaluation of traffic signal control algorithms.	Accurate measurement of execution times.
Skordylis *et al.* [34]	Data spreading algorithms (D-Greedy, D-min cost) for optimizing the data delivery and the data acquisition.	High packet delivery ratio. Low delivery delay. The communication cost required for monitoring traffic is minimized.
Abishek *et al.* [35]	Adaptive traffic flow algorithm.	Congestion is reduced at the traffic signal. Increased utilization of the infrastructure.
Eren *et al.* [36]	ZigBee based wireless system to assist traffic flow on urban roads.	Smooth traffic flow. Lower end-to-end delays.
Laisheng *et al.* [37]	Traffic random early detection (TRED) algorithm for real-time scheduling of traffic.	Reduce Congestion.

Skordylis *et al.* [34] proposed packet-forwarding algorithms for vehicular network scenarios. They introduced two routing algorithms: Delay-bounded Greedy forwarding (D-Greedy) and Delay-bounded Minimum cost (D-Min) forwarding algorithms for minimizing the communication costs required for monitoring traffic. They conducted an experimental evaluation of proposed schemes using a discrete event simulation environment and compared the results with epidemic schemes. The proposed algorithms have outperformed in the point of communication cost as maintaining the utmost packet delivery ratio and least delivery delay.

Abishek *et al.* [35] proposed a WSN-based solution for Indian city traffic. Their aim was to make the traffic signals adapt to the dynamic traffic flow. The proposed system is simulated using the C++ language. They found that the existing infrastructure can handle 7% more vehicles.

Eren *et al.* [36] investigated the use of Programmable Logic Controllers (PLCs) and supervisory control and data acquisition (SCADA) systems in traffic control applications. ZigBee technology is used for communication between the PLC and SCADA systems. The master SCADA unit processes the collected data and synchronize traffic lights for the smooth flow of vehicles. The results from their study showed that the traffic lights can be synchronized by the PLC/SCADA system. 

Xiao *et al.* [37] proposed a Traffic Random Early Detection (TRED) algorithm for real-time traffic scheduling and to solve congestion problems. All representative traffic congestion avoidance approaches that were analysed in detail are highlighted in Table 6, sorted by author names and reference numbers.

### 4.4. Priority-Based Traffic Management Schemes

**Figure 7:**
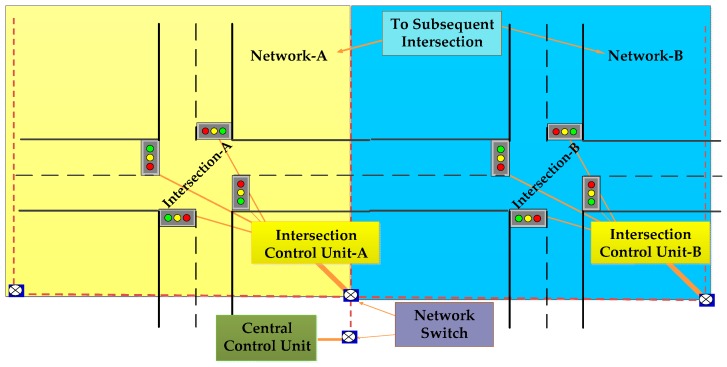
Priority based intelligent traffic management system.

An emergency vehicle is any vehicle that is designated and authorized for emergency service (such as police, ambulance and fire). Priority-based signalling will give priority to emergency vehicles and can thus save lives and property. Several WSN-based traffic management schemes and methodologies have been designed and developed to prioritize emergency vehicles and have been reported in the literature. It can be found that most of the efforts are related to intelligent traffic control system design for smooth passage of emergency vehicles [38,39,40,41,42]. The priority based intelligent traffic management system is shown in Figure 7. 

The sensors placed on traffic signals will detect the RF signal and the code transmitted from the vehicles. The sensor sends the data to the traffic light controller. The controller checks for priority and manages the signalling. After the passage of an emergency vehicle, the traffic control system reverts back to its normal operation. If two emergency vehicles of the same priority arrive at the intersection point at the same time, then signalling of the green light is given based on distance measurements of emergency vehicles from the intersection point. A vehicle with less distance from the intersection point will be given priority [43].

Farheena *et al.* [44] proposed an approach for the management of traffic systems and altering it for emergency vehicles. They presented IR and GPS methods for estimation of the traffic density and classification of an emergency vehicle. The proposed system consists of two parts: The Smart Traffic Light Control system (STLC) and the Smart Congestion Avoidance (SCA) system. The STLC system is responsible for the signalling at intersections and gives priority to emergency vehicles. The SCA system provides the best and shortest route to avoid congestion. 

An intelligent transportation system based on SIP/ZigBee architecture was proposed in [45,46] to achieve higher cost performance. The authors presented the use of ZigBee and WSNs in the metropolitan public transportation system. Their work was guided by the expert group of the ITSIC and sponsored by the Science and Technology Innovation Foundation of Hubei Province Department of Education (No: 2006JOOl).

Bottero *et al.* [47] investigated the use of WSNs for traffic monitoring. They installed the wireless sensor network for monitoring traffic in a logistic area and analysed its performance in different traffic situations.

Brahmi *et al.* [48] investigated the MAC layer in WSNs. They proposed a real-time data acquisition scheme (improved back-off selection scheme for the IEEE 802.15.4 protocol) for the faster notification of emergency events to the traffic management system. By the quick response of TMS, collision of vehicles, loss of human lives and traffic congestion are mitigated. They evaluated the end-to-end transmission delay of an incident report by using an NS-2 simulator. The simulation results proved the effectiveness of the proposed scheme in terms of faster transmission of incident messages.

The traffic signal monitor interface has been designed in Lab VIEW [49] and adopted GPRS technology to control traffic signals. All representative priority based traffic management approaches that were analysed in detail are highlighted in Table 7, sorted by author names and reference numbers.

**Table 7:** Summary of priority-based traffic management schemes.

Reference	Proposed Approach	Outcome
Rajeshwari *et al.* [38] Sireesha *et al.* [39] Shruthi *et al.* [40] Hussian *et al*. [41] Nabeel *et.al*. [42]	Implemented traffic control system.	Smooth traffic flow. Emergency vehicle clearances. Stolen vehicle detection.
Chakraborty *et al*. [43]	Real-time optimized traffic management algorithm.	Effective management of high prioritized vehicles.
Farheena *et al.* [44]	Traffic light control system and congestion avoidance systems are proposed.	Priority based signaling. Smooth traffic flow. Saving fuel consumption.
Zhou *et al*. [45] Tao *et al*. [46]	SIP/ZIGBEE based architecture for distributed traffic monitoring.	Remote communications and control operations of ITS distribution nodes are unified and simplified.
Bottero *et al*. [47]	Magnetic sensor based traffic monitoring in logistic centers.	Accurate vehicle classification and count. Low error rate.
Brahmi *et al*. [48]	Enhanced back-off section scheme for IEEE 802.15.4.	Transmission delay reduction. Faster transmission of emergency messages reporting dangerous events.

### 4.5. Average Waiting Time Reduction Schemes

Average waiting time refers to the amount of time a vehicle is waiting in a queue before the traffic signal is ON. This factor increases the travel time and shows a negative impact on transport economy. To reduce average waiting time, several WSN-based schemes and methodologies have been developed and stated in the literature. 

**Figure 8:**
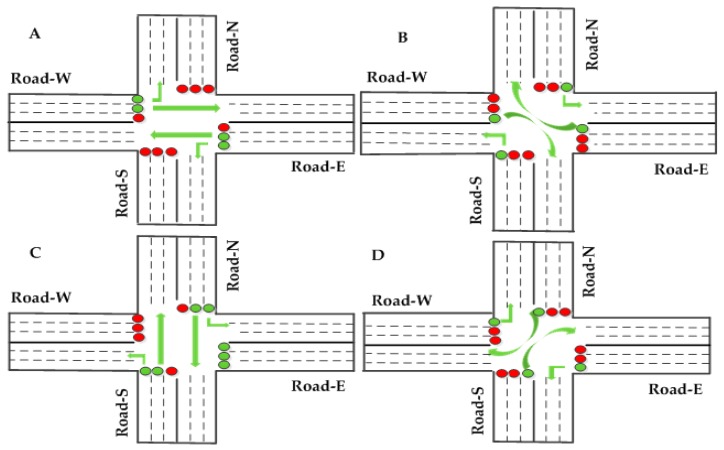
Maximum intersection utilization configuration [50].

**Figure 9:**
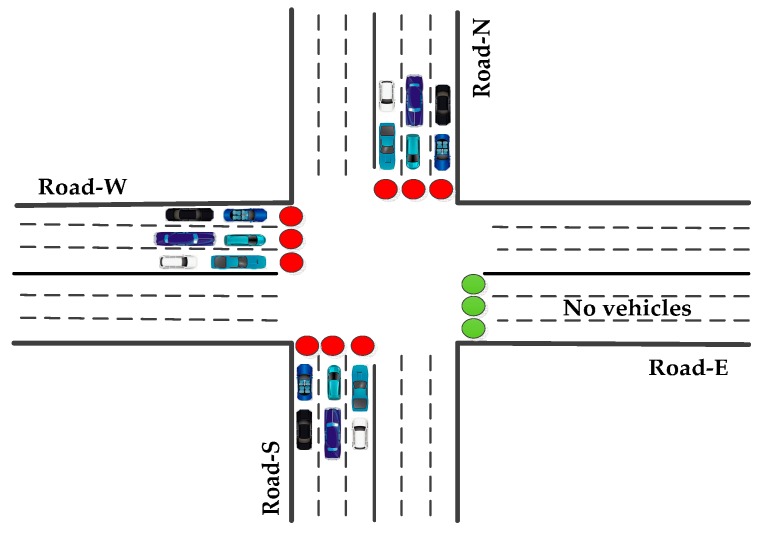
Empty lane with green light [50].

Srivastava *et al.* [50] proposed two adaptive traffic light control algorithms, *i.e.*, the maximum intersection utilization and the empty lane with green light. The maximum intersection utilization configuration is shown in Figure 8. The empty lane with green light configuration is shown in Figure 9. They tested the proposed algorithms on the Green Light District (GLD) simulator. Simulation results show that the Average Waiting Time (AWT) of the conventional policy is 26.7 cycles of wait, 22.6 cycles for maximum intersection utilization and 6.5 cycles for an empty lane with green light.

Zhou *et al.* [51] proposed an adaptive traffic light control algorithm for real-time traffic. The proposed algorithm adjusts the length and sequence of traffic lights. The proposed algorithm checks for special circumstances, blank circumstances, Hunger Level (HL), Waiting Time (WT) and Traffic Volume of the lane (TV) and then issues the signalling. The experimental outcomes showed that the proposed algorithm produces the reduction of average waiting time of vehicles and high throughput compared to fixed-time control algorithms.

**Figure 10:**
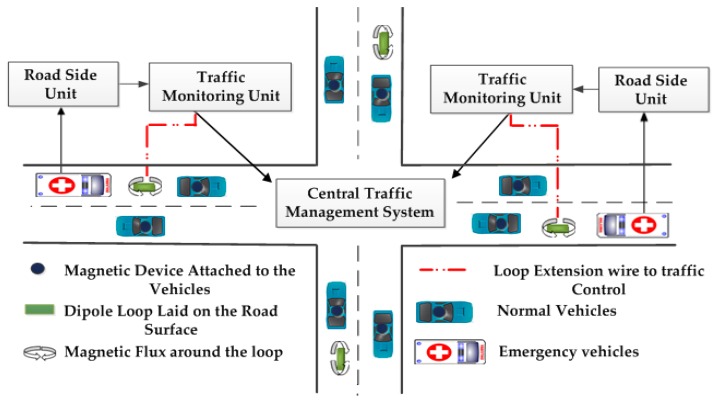
Layout architecture for efficient dynamic traffic control system [55].

The approach to make the traffic light adapt to traffic flow are discussed in [52,53,54]. Bharadwaj *et al.* [55] proposed an Efficient Dynamic Traffic Control System (EDTCS) to save vehicle traveling time and assign the highest priority for emergency vehicles at intersections. The proposed EDTCS system consists of a Traffic Control Unit (TCU), Traffic Monitor Unit (TMU) and Road Side Unit (RSU). Figure 10, shows the process at the intersection. Based on RFID tags, the emergency vehicles are identified and all the vehicles are counted by the inductive loop method. Emergency vehicles communicate with the RSU via RFID tags. If the RFID tag is genuine, the emergency vehicle count is incremented by one. The centralized traffic server collects the count of normal and emergency vehicles and toggles the signal to green to about 30 s for the side with emergency vehicles. The proposed model has solved many traffic problems and saved the traveling time. A UHF RFID tag antenna has been utilized as a displacement sensor in [56] and the authors found that the sensor is sensitive to displacements for a dynamic range of 40 mm.

**Figure 11:**
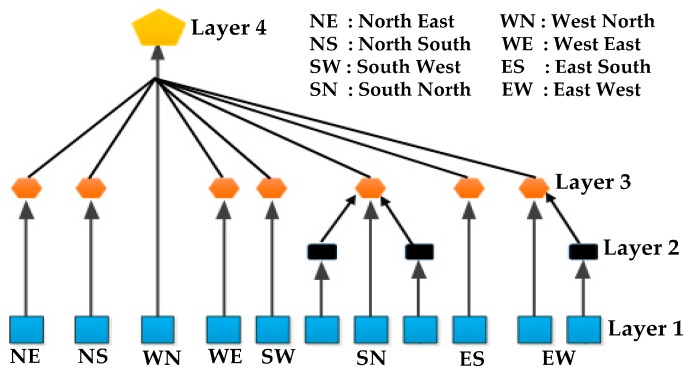
Logically separated 4-level hierarchical distributed network [57].

Faye *et al.* [57] proposed a distributed algorithm for traffic light control. They organized sensors in a hierarchical architecture. A logically separated 4-level hierarchical distributed network is shown in Figure 11. Any node can perform light plan computation. Sensors are organized in two groups: 

After-Light (AL) sensors and Before-the-Light (BL) sensors. Vehicle arrival data is continuously collected by BL sensors and departure data is collected only for the light is green by AL sensors. AL sensors are in charge of data aggregation and for taking decisions. As the sensor device directly communicates with the base station, spatial reuse and channel capacity problems are prevented. The proposed algorithm is flexible in managing conflicts and decisions. However, the authors haven’t adopted a low power mechanism to reduce the WSN node power consumption.

Dynamic traffic management techniques to reduce accidents due to the Red-Light-Running (RLR) phenomenon are discussed in [58,59,60]. The RLR phenomenon is an unhealthy and dangerous driving act. As the drivers wait in the traffic light queue, many drivers try to cross the intersection when the traffic light changes from green to yellow. This causes accidents and traffic congestion. The proposed techniques try to reduce RLR phenomenon by assigning green times to the road sections with long queues.

**Figure 12:**
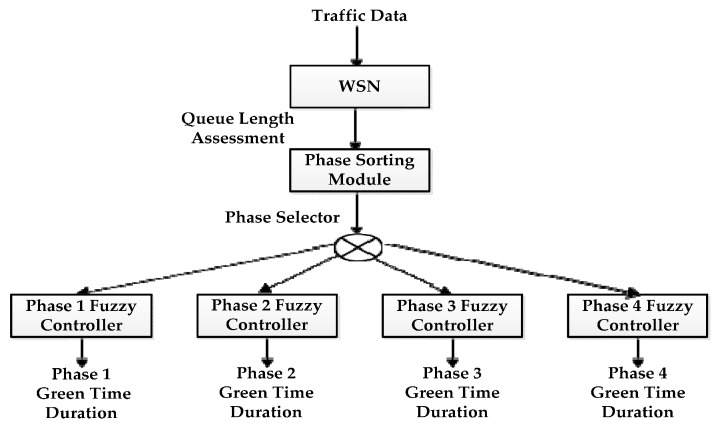
Fuzzy logic based multi controller system [61].

Some interesting methods are related to the dynamic management of traffic light cycles and phases using WSN and multi fuzzy logic controllers [61,62]. A fuzzy logic-based multi controller system is shown in Figure 12. The multi controller system consists of a Wireless Sensor Network (WSN), phase sorting module and fuzzy logic controller for each traffic light phase. The WSN acquires traffic data. A phase sorting module calculates the phase order and a fuzzy logic controller calculates the green time duration of each phase. The results demonstrate that the multi-controller approach is effective in balancing the vehicles waiting time in queues for heavy and random traffic arrivals. However, these approaches haven’t considered the priority for emergency vehicles. A robust system based on a Hidden Markov Model (HMM) can be found in [63]. The authors obtained 90.55% accuracy in the detection of the traffic light state with the proposed system. All representative average waiting time reduction approaches that were analyzed in detail are highlighted in Table 8, sorted by author names and reference numbers.

**Table 8:** Summary of average waiting time reduction schemes.

Reference	Proposed Approach	Outcome
Srivastava *et al*. [50]	Adaptive traffic flow algorithms Maximum intersection utilizations (MIU). Empty lane with green light (ELWGL).	The average waiting time: Orthodox policy: 26.7 cycles. MIU: 22.6 cycles. ELWGL: 6.5 cycles.
Zhou *et al*. [51]	Adaptive traffic light control algorithm.	Optimal green light length and green light sequence. Higher throughput. Low vehicle waiting time.
Bhuvaneswari *et al.* [52] Zhou *et al*. [53]	Adaptive traffic signal flow control system.	The system is self-configurable. Average waiting time of vehicles reduces. Detects real-time traffic stats.
Bharadwaj *et al*. [55]	Vehicle count calculation and single toggle algorithm.	Dynamic traffic light control. Reduces congestion. Saves travel time. Special priority for emergency vehicles.
Faye *et al*. [57]	Distributed algorithm to control traffic lights in urban areas.	Reduced average waiting time at an intersection. Frequent traffic light decisions.
Al-Nasser *et al*. [58] Collotta *et al*. [59]	Smart traffic signal control algorithms.	Minimized Average waiting time. Reduce the RLR phenomenon occurrence.
Collotta *et al.* [61] Wu *et al*. [62]	Dynamic traffic light control system based on WSNs and FUZZI logic controllers.	Reduces the vehicles waiting times. Real-time traffic monitoring.
Gomez *et al*. [63]	Traffic light state estimation using hidden Markov models.	Obtained 90.55% of accuracy in the detection of traffic light state.

**Figure 13:**
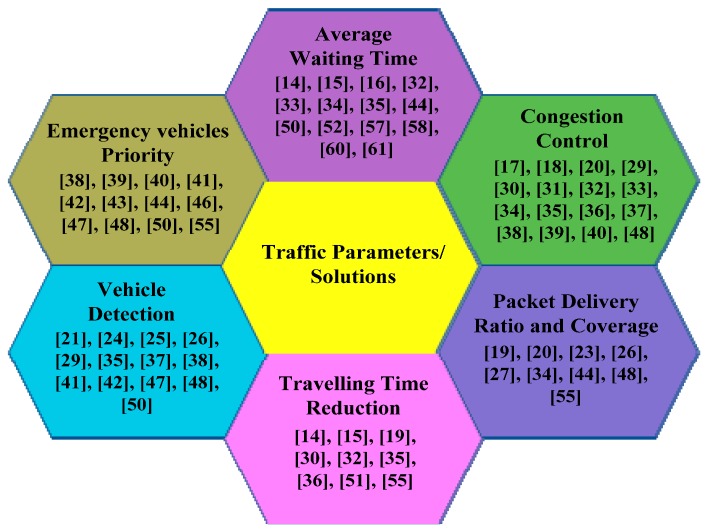
Summary of traffic parameters and solutions.

There has been considerable research on Traffic Management System using WSNs for congestion avoidance, prioritizing emergency vehicles and Average Waiting Time (AWT) reduction of vehicles at intersections. It was found that most of the developed systems were only tested in laboratory settings and only a few of these have actually been deployed in a real traffic environment [5,6,7,8,9,10,11,12,13]. Researchers have concentrated on different traffic parameters to optimize traffic management. Some of the most significant solutions that optimized traffic management and achieved parameters are shown in Figure 13.

## 5. Challenges

A WSN-based traffic management system has reduced travel time of metropolitan traffic by enhancing safety with the purpose of refining our everyday life. Traffic management systems experience some significant challenges and issues as discussed below:

*Connectivity and coverage*: Connectivity and coverage are the two vital factors for certifying effective resource management in WSNs. When many nodes are deployed to have more coverage, the efficient coordination between nodes and the central system is a big challenge. What are the confines on the number of nodes for a certain percentage of coverage?

*Communication and energy cost*: Minimizing communication cost of the system is essential to save energy and extend the network lifetime. The reliability of the system is always affected by the battery life issue. How to make use of low power mechanisms to reduce the WSN node power consumption?

*Congestion*: Despite many techniques to avoid congestion problem, the traffic management system is still not able to react quickly to non-recursive congestion. How to design a traffic management system to provide a solution for the non-recursive congestion problem?

*Traffic incident notification*: How making the traffic management system capable of sending incident notification to local media, police department and situation management office to act upon the situation?

*Coordination and implementation*: How to maintain coordination between intersections? How to design an intelligent traffic cloud to resolve real-time problems by making use of cloud computing?

## 6. Conclusions and Future Work

In this paper, we have presented a comprehensive review of existing urban traffic management schemes. The main challenges associated with congestion control, average waiting time reduction, prioritizing emergency vehicles and the design requirements of intelligent traffic system are discussed to provide an insight into the goals of urban traffic management.

Despite the large number of research activities and the excellent progress that has been made in traffic management systems in recent years, challenges for further research remains. A few issues are outlined for future work.

A real-time traffic management system cannot be guaranteed. Processing of large amounts of real time traffic data, the run time of the control system and reliability are the problems to be solved to ensure real-time demand of the urban traffic management system. There is a need to design an intelligent traffic cloud by making use of cloud computing to solve the problems related to real-time. 

The use of virtual strips in DTMon [14] can be extended for detecting and tracking of the End-of-queue, caused by congestion. Intra-vehicle channel interference [28] can be reduced further by assigning non-overlapping channels to RSUs. Modifying the dynamic traffic management system using WSN and multi fuzzy logic controllers proposed in [61] to detect emergency vehicles is an option to consider. Usage of PLCs and SCADA system in intelligent transportation system for smooth traffic flow can be an interesting issue for future research. Designing a promising traffic management system to provide smooth traffic flow in non-recursive congestion situation can be an interesting issue for future research.

In further research, it is recommended that information variables, such as the number of accidents and traffic violations are to be involved in traffic management system to assist decision makers in the formulation of traffic rules and policies.
